# Case Report: Individualized management of tuberous sclerosis complex-associated lymphangioleiomyomatosis

**DOI:** 10.3389/fmed.2026.1837160

**Published:** 2026-06-04

**Authors:** Gang Wang, Li Yang, Shiqing Qian, Sheng Hu

**Affiliations:** The Fourth Affiliated Hospital of Zhejiang University School of Medicine, Yiwu, Zhejiang, China

**Keywords:** case report, cystic lung disease, genetic screening, reproductive-age women, sirolimus, TSC-LAM

## Abstract

Tuberous sclerosis complex-associated lymphangioleiomyomatosis (TSC-LAM) is a rare systemic disorder characterized by smooth muscle-like cell proliferation, leading to cystic lung destruction, predominantly affecting women of reproductive age. We report a female patient in her mid-30s who presented with recurrent spontaneous pneumothoraces temporally associated with menstruation, diffuse bilateral pulmonary cysts on high-resolution computed tomography, bilateral renal angiomyolipomas, and facial angiofibromas. Genetic testing confirmed TSC-LAM. The patient was initiated on sirolimus (1 mg/day) with therapeutic drug monitoring to maintain trough levels within the target range. Regular follow-up included pulmonary function tests, serial imaging, and genetic counseling with cascade screening for offspring. After treatment, the disease remained clinically stable without significant adverse effects; imaging demonstrated no progression of pulmonary cysts and a reduction in renal angiomyolipoma volume. This case underscores the importance of early recognition, individualized sirolimus dosing to balance efficacy and toxicity, and systematic family screening for early detection and prevention of disease transmission in this heritable condition.

## 1 Introduction

Lymphangioleiomyomatosis (LAM) is a rare, slowly progressive systemic disease associated with cystic lung destruction, abdominal tumors, and chylous effusions caused by tumorous LAM cell infiltration (1). Tuberous sclerosis complex (TSC) is inherited as an autosomal-dominant trait and arises from germline pathogenic and likely pathogenic variants resulting in protein inactivation of *TSC1* at 9q34 or *TSC2* at 16p13 (2). LAM occurs in 34–39% of women with TSC and is then designated Tuberous sclerosis complex-associated lymphangioleiomyomatosis (TSC-LAM) (3, 4); it may also arise sporadically. We report a classic TSC-LAM case diagnosed in the reproductive years. Long-term follow-up and complete serial imaging document the benefits of individualized therapy, and we share the diagnostic pathway, treatment decisions, and surveillance plan that produced this excellent outcome.

## 2 Case description

A 36-year-old female presented to our department with a severe right-sided pneumothorax. Her medical history was notable for systemic manifestations of TSC since childhood, characterized by recurrent facial angiofibromas and periungual fibromas (Figure 1A). Ten years prior to the current admission, she underwent a left nephrectomy for a histologically confirmed renal angiomyolipoma (AML), at which time a concomitant AML was also identified in the contralateral right kidney.

**Figure 1:**
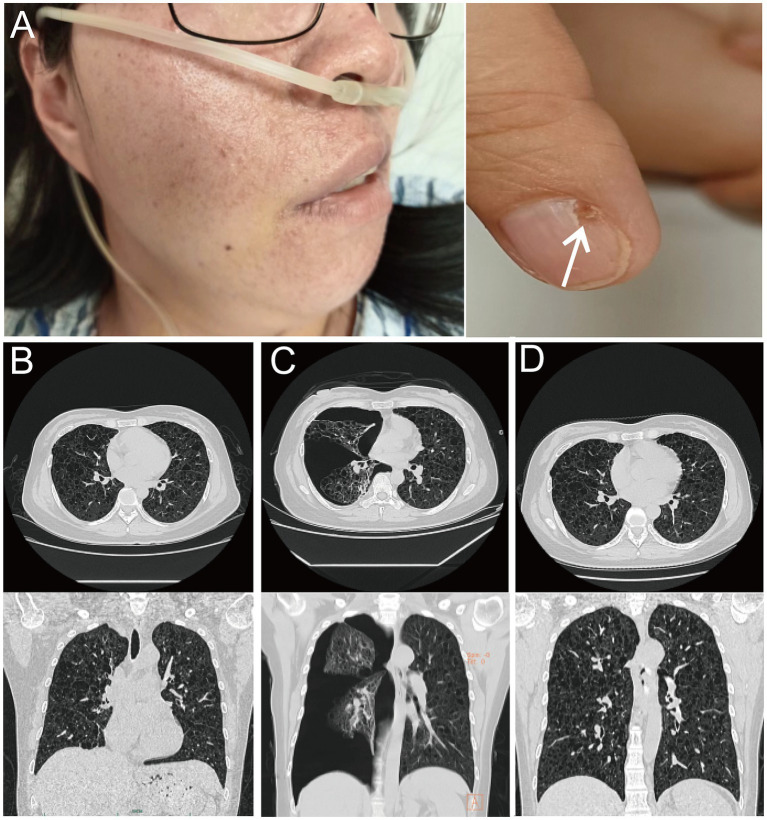
Imaging findings and clinical manifestations of the patient. **(A)** The patient exhibits recurrent facial rashes and nail fibromas; **(B)** lung CT scan taken 4 years ago, showing diffuse cystic lesions in the lungs; **(C)** 3 years ago, the patient developed a right pneumothorax on day 3 of her menstrual cycle; **(D)** lung CT scan taken at discharge 3 years ago.

Her respiratory symptoms initially manifested 8 years ago as exertional chest tightness and pain. Initial computed tomography (CT) at a local hospital revealed a pneumothorax, which was managed with needle aspiration and chest tube drainage. Over the subsequent 5 years, the patient experienced more than 10 recurrences of pneumothorax (approximately 2–3 episodes per year), demonstrating a distinct temporal correlation with her menstrual cycle (typically occurring on days 3–5 of menses). High-resolution CT (HRCT) performed 4 years ago demonstrated diffuse bilateral pulmonary cysts, the largest measuring approximately 5 cm (Figure 1B), necessitating pleurodesis.

Three years ago, on day 3 of her menstrual cycle, she developed a right-sided pneumothorax with 40% pulmonary collapse, which served as the triggering event for her admission (Figure 1C). Following emergency tube thoracostomy at our emergency department, she was transferred to our department for subsequent comprehensive management. Throughout the disease course, the patient denied symptoms of xerostomia, arthralgia, or Raynaud’s phenomenon. Laboratory investigations upon admission, including antinuclear antibodies (ANA), anti-neutrophil cytoplasmic antibodies (ANCA), immunoglobulins, complement levels, and erythrocyte sedimentation rate (ESR), were all within normal limits.

Recognizing the multisystemic nature of TSC, we performed a thorough evaluation to screen for manifestations beyond the pulmonary and renal systems. Neurological and neuropsychiatric assessments demonstrated a normal developmental trajectory and intact cognitive function. Furthermore, the patient denied any history of seizures, chronic headaches, or behavioral issues indicative of TSC-associated neuropsychiatric disorders (TAND). A contrast-enhanced brain magnetic resonance imaging (MRI) was performed to screen for intracranial anomalies; the scan was unremarkable, showing no evidence of subependymal giant cell astrocytomas (SEGAs), cortical tubers, or additional dysplasias. Furthermore, an ophthalmologic examination and a comprehensive gynecological evaluation (including pelvic ultrasound) were conducted, both of which yielded entirely normal results, effectively ruling out retinal lesions and reproductive system involvement.

Based on the differential diagnosis of air-containing lung lesions, the patient was considered to have pulmonary cysts (5). Following the diagnostic algorithm for diffuse cystic lung disease, lymphangioleiomyomatosis (LAM) was suspected. In women with chest computed tomography findings consistent with cystic lung disease, a diagnosis of LAM can be established without lung biopsy if any of the following criteria are met: ① tuberous sclerosis complex (TSC); ② serum vascular endothelial growth factor-D (VEGF-D) ≥ 800 pg/mL; ③ renal angiomyolipoma; or ④ lymphangioleiomyoma or chylous effusion (6). In this patient, the diagnosis of LAM was confirmed. The patient presented with cutaneous angiofibromas. In conjunction with the findings from skin and renal pathology, tuberous sclerosis complex (TSC) with associated LAM was suspected. The *TSC2* subtype is more common, and genetic testing was recommended for both the patient and her offspring. Subsequent genetic testing confirmed a sirolimus variant in the *TSC2*, leading to a definitive diagnosis of TSC-associated LAM. The patient was treated with sirolimus at a dose of 1 mg/day, with symptomatic improvement observed after treatment (Figure 1D).

Post-discharge, follow-up visits were scheduled every 3 months. Sirolimus trough concentrations were maintained between 3 and 5 ng/mL. Disease remained stable, with no drug-related adverse events. Over the next 3 years, only a single, small, right-sided pneumothorax occurred 5 months after starting therapy; lung collapse was <10% and resolved without intervention (Figure 2A). Serial high-resolution CT demonstrated no progression of pulmonary cystic disease, while abdominal CT revealed a progressive decrease in volume of the right renal angiomyolipoma (Figures 2B,C). Furthermore, routine neurological evaluations conducted during these visits remained entirely unremarkable, with no emergence of seizures, chronic headaches, or cognitive changes. The 6-min walk test distance improved from 367 meters at discharge to 497 meters at the one-year follow-up evaluation. However, due to the patient’s history of high-frequency recurrent pneumothoraces and the potential risk of triggering a recurrence during forced expiratory maneuvers, serial pulmonary function tests (PFTs) were not performed during the follow-up period. The respiratory status was instead monitored via serial high-resolution CT imaging, stable resting oxygen saturation, and exercise tolerance.

**Figure 2:**
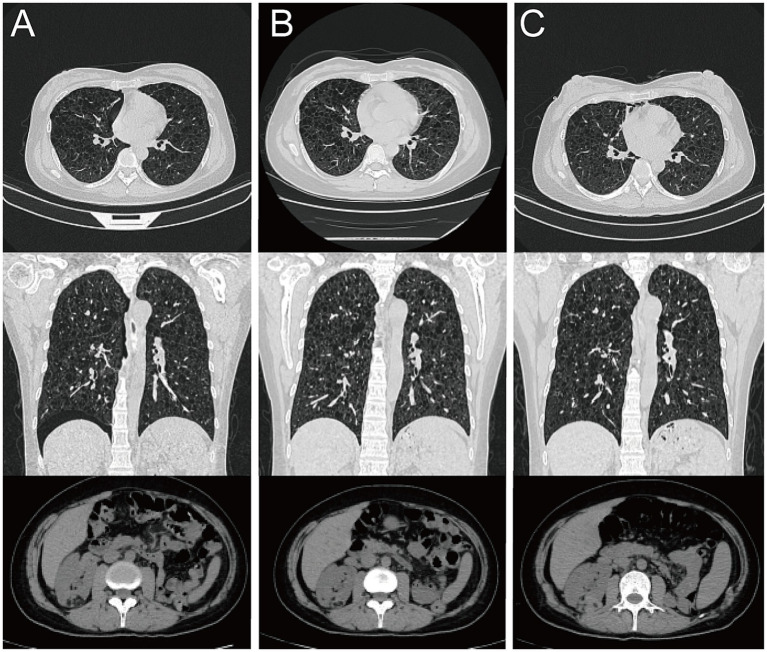
Imaging studies during follow-up. **(A)** Five months after initiating sirolimus therapy (two and a half years ago), the patient experienced the last spontaneous pneumothorax, which was only 10% in volume and resolved spontaneously; **(B, C)** Follow-up chest and abdominal CT scans performed 1 year ago and 1 month ago, respectively, showed no significant progression on chest CT and a reduction in the size of the right renal angiomyolipoma.

## 3 Discussion

LAM is a rare, progressive multisystem disorder in which abnormal smooth-muscle–like cells (LAM cells) infiltrate the lung, producing cystic parenchymal destruction, lymphatic abnormalities, and renal angiomyolipomas (7). TSC is an autosomal dominant genetic disorder that, beyond pulmonary involvement, can affect multiple organ systems, including the skin, kidneys, and central nervous system, manifesting as benign tumors or structural malformations; its primary clinical manifestations and diagnostic workflow are summarized in Table 1. Notably, the central nervous system (CNS) is a primary site of involvement in TSC, manifesting as structural lesions—such as subependymal giant cell astrocytomas (SEGAs)—and a spectrum of TSC-associated neuropsychiatric disorders (TAND) (8). Although our patient’s neurological evaluation and brain MRI were unremarkable, documenting these negative findings highlights the significant phenotypic variability of TSC. It underscores that comprehensive neurocutaneous and neuropsychiatric screening is mandatory even for patients presenting primarily with respiratory symptoms, ensuring early detection of potentially life-threatening CNS complications.

**Table 1:** Clinical diagnostic criteria for tuberous sclerosis complex.

Major features	Minor features
1. Hypomelanotic macules (≥3, at least 5 mm diameter)	1. “Confetti” skin lesions
2. Angiofibroma (≥3) or cephalic fibrous plaque	2. Dental enamel pits (≥3)
3. Ungual fibromas (≥2)	3. Intraoral fibromas (≥2)
4. Shagreen patch	4. Retinal achromic patch
5. Multiple retinal hamartomas	5. Multiple renal cysts
6. Multiple cortical tubers and/or radial migration lines	6. Nonrenal hamartomas
7. Subependymal nodule (≥2)	7. Sclerotic bone lesions
8. Subependymal giant cell astrocytoma	
9. Cardiac rhabdomyoma	
10. LAM	
11. Angiomyolipomas (≥2)	

The combination of LAM and angiomyolipoma alone, in the absence of additional features, does not fulfill the criteria for a definite diagnosis.

Based on the presence or absence of TSC, LAM is classified into sporadic LAM and TSC-LAM. TSC-LAM results from pathogenic or likely pathogenic variants in either the *TSC1* or *TSC2*, leading to dysregulated cellular proliferation, aberrant angiogenesis, and lymphatic dysplasia, thereby promoting LAM cell infiltration and cystic lesion formation (9). Epidemiologically, TSC-LAM predominantly affects women of reproductive age (20–40 years), with an earlier age of onset compared to sporadic LAM (mean age 25–35 years). Among female TSC patients, 30–60% develop LAM-associated cystic pulmonary changes, whereas the prevalence of cystic lung lesions in male TSC patients ranges from only 10 to 38%, with the majority remaining asymptomatic (10).

The pathogenesis of LAM is intrinsically hormone-dependent, underpinning its marked predominance in women of reproductive age. Clinically, hormonal fluctuations directly dictate the disease trajectory: pregnancy or exogenous estrogen administration can precipitate a rapid decline in pulmonary function, whereas disease progression typically attenuates post-menopause. At the cellular and molecular levels, LAM cells highly express estrogen and progesterone receptors (7). Through these receptors, estrogen actively promotes cellular survival and metastatic dissemination by stimulating the MEK-MAPK and Akt signaling pathways, thereby driving the accelerated disease progression observed during the reproductive years (11).

The central pathogenetic mechanism of LAM involves pathogenic or likely pathogenic variants in the *TSC1* and *TSC2*, leading to loss of function of the hamartin-tuberin complex and subsequent hyperactivation of the mTORC1 signaling pathway (12). This constitutive activation promotes aberrant cellular proliferation, angiogenesis, and lymphangiogenesis—mediated via upregulation of vascular endothelial growth factor (VEGF)-A, VEGF-C, and VEGF-D—ultimately resulting in cystic lesion formation (13). LAM exhibits metastatic potential, with LAM cells expressing smooth muscle actin (SMA) and melanoma-specific antigen HMB-45 and demonstrating bidirectional smooth muscle-melanocytic differentiation features—potentially originating from extrapulmonary sites such as the uterus and capable of recurring in allograft lungs following transplantation (14).

The clinical manifestations of this disease predominantly include exertional dyspnea (50%) and spontaneous pneumothorax (30%), with symptoms in female patients frequently correlating with the menstrual cycle (15); the latter is notable for an exceptionally high relapse rate. HRCT shows evenly distributed, thin-walled cysts 2–30 mm in diameter separated by normal lung parenchyma (16). Cutaneous angiofibromas are common, and up to 80% of patients harbour bilateral renal angiomyolipomas (AMLs) that carry a substantial risk of haemorrhage once they exceed 4 cm. Definite LAM is diagnosed when characteristic HRCT cystic changes are accompanied by any one of the following: (1) definite TSC, (2) serum VEGF-D ≥ 800 pg/mL, or (3) renal AML (17). Our patient fulfilled two of these specific criteria (the definitive presence of TSC and concomitant bilateral renal AMLs). Although serum VEGF-D levels were not assessed—which we acknowledge as a limitation of our retrospective clinical data—the combination of her radiological findings and multi-organ manifestations unequivocally fulfilled the threshold for a definitive diagnosis of TSC-LAM.

Given the presentation of recurrent pneumothoraces and multisystem involvement, a rigorous differential diagnosis of diffuse cystic lung diseases (DCLDs) is imperative. Birt-Hogg-Dubé (BHD) syndrome was excluded by the characteristic morphology of the pulmonary cysts—which were diffuse and round rather than the basal-predominant, lentiform cysts typical of BHD—and the presence of benign renal AMLs instead of malignant carcinomas (18). Langerhans cell histiocytosis (LCH) was deemed unlikely given the patient’s non-smoking status and the absence of upper-lobe predominant stellate cysts or nodules (19). Lymphocytic interstitial pneumonia (LIP) and light chain deposition disease (LCDD) were further ruled out by the lack of associated autoimmune features or monoclonal gammopathy.

Notably, the temporal correlation between the pneumothoraces and the patient’s menstrual cycle initially raised suspicion for Thoracic Endometriosis Syndrome (TES) (20). However, while TES can cause catamenial pneumothorax, it typically lacks the diffuse parenchymal cysts observed on this patient’s HRCT. The “catamenial-like” presentation in this case is better explained by the hormone-sensitive nature of LAM cells. Furthermore, although Neurofibromatosis type 1 (NF1) is a major neurocutaneous syndrome that can involve the lungs, it typically manifests as upper-lobe bullous or emphysematous changes alongside café-au-lait macules and neurofibromas, which were absent here (21). The definitive diagnosis of TSC-LAM was ultimately solidified by the pathognomonic combination of facial angiofibromas, bilateral renal AMLs, and the identification of the germline *TSC2* mutation.

First-line therapy is an mTOR inhibitor (sirolimus or everolimus). The landmark Multicenter International LAM Efficacy of Sirolimus (MILES) trial robustly established the efficacy and safety of sirolimus, demonstrating its ability to stabilize lung function and improve symptoms in LAM patients (22). Furthermore, these agents block aberrant mTORC1 signaling and induce regression of renal AMLs, thereby improving long-term quality of life (23).

The cornerstone of our individualized management strategy was the dynamic titration of sirolimus, aimed at balancing therapeutic efficacy against the risk of nephrotoxicity in a patient with a solitary kidney. While the conventional sirolimus trough range for LAM is historically established at 5–15 ng/mL, this patient’s baseline renal reserve was inherently vulnerable due to her status post-left nephrectomy and the presence of multifocal angiomyolipomas (AMLs) in the remaining right kidney. Our clinical decision-making followed the ‘lowest effective dose’ principle. We deliberately targeted a sub-guideline trough level of 3–5 ng/mL. This localized threshold was informed by early therapeutic drug monitoring, which revealed that even concentrations approaching the standard lower limit (5 ng/mL) were associated with transient elevations in serum creatinine (Figure 3).

**Figure 3:**
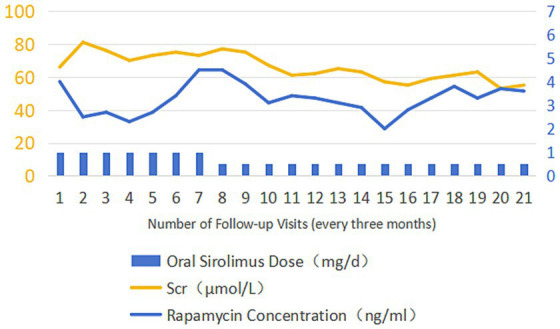
Plasma drug concentration and serum creatinine concentration during follow-up.

By maintaining this ‘sub-therapeutic’ range, we achieved a successful longitudinal equilibrium: the disease remained clinically stable with no recurrence of pneumothorax over a three-year follow-up period, while the volume of the right renal AML significantly decreased and the estimated glomerular filtration rate (eGFR) remained strictly stable. This individualized trade-off underscores that in compromised anatomical contexts, clinical outcomes can be optimized by tailoring drug exposure below standard conventional thresholds.

Current comprehensive follow-up protocols recommend annual HRCT plus spirometry, six-monthly renal ultrasonography, periodic brain MRI alongside clinical neurological evaluations, and serial measurement of sirolimus trough concentrations and serum VEGF-D. Furthermore, surgical or chemical pleurodesis lowers the pneumothorax recurrence rate from ~70% to ~30% (24).

Considering that TSC-LAM is inherited in an autosomal-dominant manner, we performed cascade testing for the patient’s 8-year-old daughter. Prior to whole-exome sequencing, formal genetic counseling was provided to the parents by a qualified physician. This session extensively addressed the ethical and psychological implications of cascade genetic testing for an age-dependent heritable disorder in a minor. Specific written informed consent for both the counseling process and the pediatric testing was obtained and documented in the medical records, separate from the standard consent for treatment and publication.

Genetic analysis confirmed that the daughter carries the same germline pathogenic *TSC2* variant (c.1831C > T) as her mother. Clinically, she presented with recurrent facial rashes suspected to be early angiofibromas, consistent with the age-related expression of TSC (25). To rule out occult central nervous system involvement, a comprehensive neurological evaluation was subsequently conducted; the examination revealed no objective symptoms or signs, and the neurological assessment was entirely negative. However, given the multisystemic and neurogenetic nature of TSC, she has been enrolled in a comprehensive multi-disciplinary surveillance program encompassing baseline neurodevelopmental screening and brain magnetic resonance imaging to monitor for subependymal nodules or cortical dysplasias, alongside annual renal ultrasounds and pulmonary function tests to detect early signs of LAM as she reaches adolescence. Furthermore, our counseling addressed the implications for future reproductive health, including the option of preimplantation genetic testing, ensuring that the identification of this pathogenic variant facilitates proactive management and family-wide disease prevention.

## 4 Conclusion

TSC-LAM is a relatively rare and profoundly multisystemic disorder, and its variable clinical manifestations often lead to delayed diagnosis. A multidisciplinary team that integrates pulmonology, radiology, nephrology, neurology, clinical genetics, and gynaecology is essential for early recognition and comprehensive management. Any woman of reproductive age who experiences recurrent, apparently idiopathic pneumothorax should immediately undergo high-resolution CT of the chest, accompanied by systematic screening for neurocutaneous TSC stigmata and renal angiomyolipomas. Once the diagnosis is confirmed, sirolimus should be started without delay; drug concentrations should be individualized outside conventional target ranges to secure disease control while minimizing toxicity in patients with compromised renal reserve. Prompt surgical or chemical pleurodesis at the first episode of pneumothorax reduces the 70% lifetime recurrence risk by half. Finally, cascade genetic testing and longitudinal multisystem surveillance must be offered to all first-degree relatives, beginning in childhood and extending through the reproductive years.

## Data Availability

The original contributions presented in the study are included in the article/supplementary material, further inquiries can be directed to the corresponding author.

## References

[1] Xu KF Xu W Liu S Yu J Tian X Yang Y . Lymphangioleiomyomatosis. Semin Respir Crit Care Med. (2020) 41:256–68. doi: 10.1055/s-0040-1702195, 32279296

[2] Ambrosetti I Cristalli CP Montanari F Caramanna L Modestino F Capelli I . Low-level mosaicism in tuberous sclerosis complex (TSC): diagnostic and clinical implications from two novel cases and literature review. Am J Med Genet A. (2026) 200:e70018. doi: 10.1002/ajmga.70018, 41486106

[3] Moss J Avila NA Barnes PM Litzenberger RA Bechtle J Brooks PG . Prevalence and clinical characteristics of lymphangioleiomyomatosis (LAM) in patients with tuberous sclerosis complex. Am J Respir Crit Care Med. (2001) 164:669–71. doi: 10.1164/ajrccm.164.4.2101154, 11520735

[4] Gai Z He Y Zhao Q Du X Zhao J. Recent advances in understanding the pathogenesis, diagnosis, and treatment of tuberous sclerosis complex (TSC)-associated Lymphangioleiomyomatosis. Biochem Pharmacol. (2026) 246:117697. doi: 10.1016/j.bcp.2026.117697, 41519401

[5] Rebaine Y Nasser M Girerd B Leroux C Cottin V. Tuberous sclerosis complex for the pulmonologist. Eur Respir Rev. (2021) 30:200348. doi: 10.1183/16000617.0348-2020, 34348978 PMC9488995

[6] Franciosi AN Gupta N Murphy DJ Wikenheiser-Brokamp KA McCarthy C. Diffuse cystic lung disease: a clinical guide to recognition and management. Chest. (2025) 167:529–47. doi: 10.1016/j.chest.2024.08.008, 39168181

[7] Krückel A Gocke J Hörner M Keller K Müller C Brückner L . Genetic tumor syndromes in female cancer: insights into inherited cancer predisposition and clinical implications. Arch Gynecol Obstet. (2026) 313:38. doi: 10.1007/s00404-025-08270-6, 41528496 PMC12799638

[8] de Vries PJ Heunis TM Vanclooster S Chambers N Bissell S Byars AW . International consensus recommendations for the identification and treatment of tuberous sclerosis complex-associated neuropsychiatric disorders (TAND). J Neurodev Disord. (2023) 15:32. doi: 10.1186/s11689-023-09500-1, 37710171 PMC10503032

[9] Yin HQ Li XF Fu Y Zhu HL Luo YS. A case report and review of rheumatoid arthritis co-occurring with tuberous sclerosis complex, a rare occurrence. Front Immunol. (2024) 15:1425988. doi: 10.3389/fimmu.2024.1425988, 39391300 PMC11464351

[10] Cottin V Cases A Bourdin V Reynaud-Gaubert M Hirschi S Kerjouan M . Characteristics and outcomes of patients with LAM receiving sirolimus in France based on real-life data. Front Med (Lausanne). (2024) 11:1494713. doi: 10.3389/fmed.2024.1494713, 39845810 PMC11753350

[11] Cao K Song M Yu R Zhao Y Chen P Wang C . Oroxylin a suppresses pathological vascular smooth muscle cell phenotypic switching and neointima formation through hindering TSC2/mTORC1/HIF-1-dependent glycolysis. Phytother Res. (2026) 40:907–23. doi: 10.1002/ptr.70145, 41520366

[12] Verwer EE Kavanagh TR Mischler WJ Feng Y Takahashi K Wang S . [(18)F]Fluorocholine and [(18)F]Fluoroacetate PET as imaging biomarkers to assess phosphatidylcholine and mitochondrial metabolism in preclinical models of TSC and LAM. Clin Cancer Res. (2018) 24:5925–38. doi: 10.1158/1078-0432.CCR-17-3693, 30054282 PMC6816044

[13] Zahedi K Barone S Brooks M Zhang W Yu JJ Zaidman NA . The critical role of the proto-oncogene c-kit in TSC renal cystogenesis. EMBO Mol Med. (2026) 18:575–598. doi: 10.1038/s44321-025-00360-x, 41429944 PMC12905254

[14] Clements D Babaei-Jadidi R Johnson J Miller S Shah N Sand JMB . Extracellular matrix deposition drives disease progression and reduces rapamycin response in LAM. Eur Respir J. (2025) 67:2500492. doi: 10.1183/13993003.00492-2025, 41381226

[15] Liang PQ Liu J. Current status and prospects of exploring the etiology of diffuse cystic lung disease. Zhonghua Jie He He Hu Xi Za Zhi. (2026) 49:7–10. doi: 10.3760/cma.j.cn112147-20250828-00526, 41483914

[16] Torasawa M Shukuya T Uemura K Hayashi T Ueno T Kohsaka S . Lymphangioleiomyomatosis as a potent lung cancer risk factor: insights from a Japanese large cohort study. Respirology. (2024) 29:815–24. doi: 10.1111/resp.14724, 38654512

[17] McCarthy C Gupta N Johnson SR Yu JJ McCormack FX. Lymphangioleiomyomatosis: pathogenesis, clinical features, diagnosis, and management. Lancet Respir Med. (2021) 9:1313–27. doi: 10.1016/S2213-2600(21)00228-9, 34461049

[18] Patel TM Beal MA Schroeder G Shifren A. Birt-Hogg-Dubé Syndrome. Clin Chest Med. (2025) 46:619–32. doi: 10.1016/j.ccm.2025.07.004, 41110925

[19] Qanneta R Raventos-Estelle A. Pulmonary Langerhans-cell histiocytosis. N Engl J Med. (2022) 387:2449. doi: 10.1056/NEJMicm2203885, 36573701

[20] Chen ML Li CY. Thoracic endometriosis. N Engl J Med. (2021) 385:e65. doi: 10.1056/NEJMicm2105508, 34731539

[21] Wilson BN John AM Handler MZ Schwartz RA. Neurofibromatosis type 1: new developments in genetics and treatment. J Am Acad Dermatol. (2021) 84:1667–76. doi: 10.1016/j.jaad.2020.07.105, 32771543

[22] McCormack FX Inoue Y Moss J Singer LG Strange C Nakata K . Efficacy and safety of sirolimus in lymphangioleiomyomatosis. N Engl J Med. (2011) 364:1595–606. doi: 10.1056/NEJMoa1100391, 21410393 PMC3118601

[23] Rodríguez-Olivares JL González-Sánchez HR Beas-Lozano EL Arteaga-Vázquez J Elaine TLM Bourlon MT. Hereditary renal tumor syndromes and the use of mTOR inhibitors. Oncology (Williston Park). (2024) 38:376–80. doi: 10.46883/2024.25921027, 39475505

[24] Mushir A Akhtar S Hussain M. Tuberous sclerosis associated lymphangioleiomyomatosis: a case report. J Pak Med Assoc. (2023) 73:908–11. doi: 10.47391/JPMA.5546, 37052013

[25] Amin S Kingswood JC Bolton PF Elmslie F Gale DP Harland C . The UK guidelines for management and surveillance of tuberous sclerosis complex. QJM. (2019) 112:171–82. doi: 10.1093/qjmed/hcy215, 30247655

